# 非小细胞肺癌*MET*基因突变的机制及靶向药物研究进展

**DOI:** 10.3779/j.issn.1009-3419.2020.102.32

**Published:** 2020-07-20

**Authors:** 森 韩, 旭 马, 健 方

**Affiliations:** 100142 北京，北京大学肿瘤医院暨北京市肿瘤防治研究所，恶性肿瘤发病机制及转化研究教育部重点实验室 Key Laboratory of Carcinogenesis and Translational Research (Ministry of Education), Peking University Cancer Hospital & Institute, Beijing 100142, China

**Keywords:** 肺肿瘤, MET, 靶向治疗, 沃利替尼, Lung neoplasms, MET, Target therapy, Savolitinib

## Abstract

间质-上皮细胞转化因子（mesenchymal-epithelial transition factor, *MET*）基因是非小细胞肺癌（non-small cell lung cancer, NSCLC）的一种重要肿瘤驱动基因，针对MET 14外显子的跳跃突变的靶向治疗药物给患者带来新的希望。目前已经上市或者即将上市的MET抑制剂包括：克唑替尼、卡博替尼、沃利替尼和Tepotinib等。MET抑制剂的客观缓解率较高，并且安全性良好。但是，MET抑制剂的耐药不可避免，因此需要重视对于耐药机制的研究。肝细胞生长因子（hepatocyte growth factor, HGF）/MET信号通路抑制剂与其他药物的联合应用，对于抑制和逆转耐药可能发挥重要作用。

## Abstract (en)

Mesenchymal-epithelial transition factor (*MET*) gene is an important tumor driver gene of non-small cell lung cancer (NSCLC). Drugs targeting MET 14 exon skipping mutation bring new hope to patients. MET inhibitors that are currently on the market or are about to be marketed include: crizotinib, cabozantinib, savolitinib and tepotinib. The objective response rate of MET inhibitors is high, and the safety is good. However, resistance of MET-tyrosine kinase inhibitor (TKI) is inevitable, so it is necessary to pay attention to the study of drug resistance mechanism. In addition, the combined use of hepatocyte growth factor (HGF)/MET inhibitors and other drugs may play an important role in inhibiting and reversing drug resistance.

在非小细胞肺癌（non-small cell lung cancer, NSCLC）的治疗中，针对特定肿瘤驱动基因的靶向药物发挥着极其重要的作用。目前已明确的靶点有表皮生长因子受体（epidermal growth factor receptor, *EGFR*）突变、间变性淋巴瘤激酶（anaplastic lymphoma kinase, *ALK*）重排和*ROS1*重排等^[1, 2]^。针对这些靶点分别有不同种类的小分子酪氨酸激酶抑制剂（tyrosine kinase inhibitors, TKIs）。间质-上皮细胞转化因子（mesenchymal-epithelial transition factor, MET）被认为是继*EGFR*、*ALK*和*ROS1*之后在NSCLC中的另一个重要肿瘤驱动基因和治疗靶点，目前受到越来越多的关注^[3-5]^。*MET*基因的异常主要包括3种形式：*MET* 14外显子跳跃突变、*MET*基因扩增和蛋白过表达。其中，针对*MET* 14外显子跳跃突变的靶向药物最具发展和应用前景，本文中*MET*基因突变主要指*MET* 14外显子跳跃突变。目前，至少有7种针对*MET*基因突变的TKI已经上市或者正在进行临床试验，包括克唑替尼、卡博替尼、沃利替尼、Tepotinib、Capmatinib、Glesatinib和Merestinib，另外有更多的药物在进行临床前研究^[6, 7]^。本文将结合*MET* 14外显子突变的特点，重点对这些药物的研究情况和临床应用进行综述，并提出未来MET抑制剂的发展方向和面临的挑战。

## 1 *MET*基因突变

### 1.1 *MET*基因和肝细胞生长因子（hepatocyte growth factor, HGF）/MET信号通路

*MET*基因位于人类7号染色体（7q21-31），长度约125 kb，同时含有21个外显子^[8]^。由*MET*基因编码的蛋白为c-MET，也称为肝细胞生长因子受体（hepatocyte growth factor receptor, HGFR），是具有自主磷酸化活性的跨膜受体，属于酪氨酸激酶受体超家族，主要表达于上皮细胞。HGF是目前发现的c-MET的唯一配体，属于纤维蛋白溶酶原家族，主要表达于间质细胞。HGF能够与c-MET的细胞外结构域结合，促使c-MET发生二聚化、酪氨酸磷酸化，激活众多下游信号通路，如PI3K-Akt、Ras-MAPK、STAT和Wnt/β-catenin等，从而发挥促进细胞增殖、细胞生长、细胞迁移、侵袭血管及血管生成等效应。这在正常的组织发育和肿瘤进展中都发挥重要作用，即c-MET正常表达时促进组织的分化与修复，当调节异常时则促进肿瘤的增殖与转移^[9]^。c-MET通路异常激活主要包括*MET* 14外显子跳跃突变、*MET*基因扩增和c-MET蛋白过表达3种类型。

### 1.2 *MET* 14外显子跳跃突变

c-MET主要由E3泛素连接酶c-Cbl主导降解。*MET* 14外显子对应编码141个氨基酸，其所在的近膜结构域是c-MET的关键负性调控区，包含着E3泛素连接酶c-Cbl酪氨酸结合位点（Y1003），参与c-MET蛋白的泛素化和降解。*MET* 14外显子的基因突变会引起14外显子跳读（exon skipping），使得含有E3泛素连接酶c-Cbl结合位点的近膜结构域缺失，进而导致c-MET蛋白泛素化障碍、c-MET稳定性增加和降解率减低，引起下游信号的持续激活，最终成为肿瘤的驱动基因^[10]^。

在NSCLC中，*MET* 14外显子跳跃突变的总体发生率大约在3%-6%^[11, 12]^。美国癌症基因研究组通过对230例肺腺癌的mRNA和DNA高通量测序结果进行分析，发现约4%的肺腺癌存在*MET* 14外显子跳跃突变^[13]^。Awad等^[14]^利用二代测序的方法检测933例NSCLC患者的基因，发现28例（3.0%）NSCLC患者存在*MET* 14外显子跳跃突变。在肺肉瘤样癌中，*MET* 14外显子跳跃突变的发生率可能更高，达到约22%^[15]^。在肺鳞癌中，*MET* 14外显子跳跃突变的发生率较低，约为2%^[16]^。在中国人群中，其突变发生率可能明显低于高加索人群，Liu等^[17]^分析1, 296例中国NSCLC患者的DNA，发现仅有12例（0.9%）患者具有*MET* 14外显子跳跃突变，这与Zheng等^[18]^研究的结果相似（1.3%, 23/1, 770）。既往研究^[19, 20]^显示*MET* 14外显子跳跃突变不与*EGFR*、*ALK*等NSCLC的其他驱动基因共存，提示其代表一种独立的肿瘤驱动基因，但是*MET* 14外显子跳跃突变可以与*MET*基因扩增和蛋白过表达并存^[21]^。对于*MET* 14外显子跳跃突变、*MET*基因扩增和c-MET蛋白过表达三者之间是否存在必然联系，不同的研究有着不同的结果，目前尚无大规模的研究证据支持。

## 2 作用于*MET*基因突变的药物机制

基于HGF/c-Met信号通路异常激活，*Met*基因突变成为NSCLC中重要的治疗靶点。根据HGF/c-Met信号通路中作用位点的不同，可将靶向治疗药物分为三大类：抗HGF单克隆抗体、抗c-Met单克隆抗体和小分子TKI。前两者分别在细胞外与HGF和c-Met结合，从而阻止HGF与c-Met的结合及受体磷酸化，阻止信号传导；小分子TKI作用于膜内催化域从而阻止蛋白磷酸化，阻断信号传导。目前研究最多的也最具有治疗潜力的是小分子TKI，因此本文重点关注此类靶向药物。

根据作用机制，针对*MET*基因突变的TKI（MET-TKI）可分为3种类型（Ⅰ型、Ⅱ型和Ⅲ型）^[22]^。Ⅰ型TKI是ATP竞争性的，与MET主链中的氨基酸残基形成氢键，其中又分为Ⅰa型和Ⅰb型，区别在于Ⅰb型TKI可以结合的位点较少，不包括甘氨酸残基的G1163位点（类似于*ALK*的G1202和*ROS1*的G2032位点），因此特异性较高。临床常用的药物克唑替尼属于Ⅰa型MET-TKI，Tepotinib和沃利替尼属于Ⅰb型MET-TKI。Ⅱ型MET-TKI一般为多靶点TKI，不仅占据ATP结合位点，还能通过管家基因突变进入非活性DFG-out构象形成的疏水口袋，对产生二次突变的MET仍具有抑制作用，或许可以逆转由Y1230等突变引起的Ⅰ型MET-TKI耐药^[6]^。卡博替尼属于Ⅱ型MET-TKI。Ⅲ型MET-TKI作用于与ATP结合位点完全不同的变构位点，目前尚没有药物进入临床研究阶段。

## 3 作用于*MET*基因突变的靶向药物

下面将分别介绍目前已经上市、即将上市和正在进行临床试验的几种MET-TKI（表 1）。

**1:** 作用于*MET* 14外显子跳跃突变的靶向药物 TKIs targeting *MET* gene mutation

Drugs	Targets	Types of TKI	Current status	Clinical trial number	Affiliated company
Crizotinib	*MET*, *ALK*, *ROS-1*	Ia	On the market	NCT0058519 NCT02465060 NCT02499614 NCT02664935	Pfizer
Cabozantinib	*MET*, *VEGFR2*, *RET*, *KIT*, *AXL*	Ⅱ	On the market	NCT01639508	Elexis
Savolitinib	*MET*	Ⅰb	In the process of marketing	NCT02897479	Hutchison MediPharma
Tepotinib	*MET*	Ⅰb	On the market	NCT02864992/2015-005 696-24	Merck
Capmatinib	*MET*	Ⅰb	In the process of marketing	NCT02750215 NCT01324479	Novartis
Glesatinib	*MET*, *VEGFR*, *RON*, *TIE-2*	Ⅱ	Clinical trials in progress	NCT02544633	Mirati Therapeutics
Merestinib	*MET*, *TIE-1*, *AXL*, *ROS1*, *DDR1*/*2*, *FLT3*, *MERTK*, *RON*, *MKNK1*/*2*	Ⅱ	Clinical trials in progress	NCT02920996	Eli Lilly
EGFR: epidermal growth factor receptor; ALK: anaplastic lymphoma kinase; TKI: tyrosine kinase inhibitor; VEGFR2: vascular endothelial growth factor receptor 2.					

### 3.1 克唑替尼

克唑替尼（Crizotinib）是多靶点药物，其作用于*ALK*、*ROS1*、*MET*等^[23]^。目前其获批的适应证是*ALK*和*ROS-1*基因重排的NSCLC。克唑替尼作为Ⅰa型MET-TKI能够抑制c-MET的自身磷酸化，从而抑制下游信号通路、抑制细胞增殖和促进凋亡^[24]^。2020年最新版美国国家癌症综合网（National Comprehensive Cancer Network, NCCN）指南（Version 3.2020）推荐克唑替尼可以用于*MET*基因扩增和*MET* 14外显子突变的晚期NSCLC患者，这是目前NCCN指南中推荐的唯一一个MET-TKI。这一推荐是基于临床试验的结果，例如在PROFILE-1001研究中招募了21例初治或者化疗后耐药的*MET* 14外显子突变的NSCLC患者，其中18例在初步报告时可评估疗效，在中位随访时间相对较短的5.3个月内，客观缓解率（objective response rate, ORR）达到44%^[25]^。最新发表在*Nature Medicine*上的报道对PROFILE-1001进行了扩展，在69例*MET*外显子14突变的晚期NSCLC患者中评估克唑替尼的抗肿瘤活性和安全性。在65例可评估反应的患者中，ORR为32%（95%CI: 21%-45%）^[26]^。因此，克唑替尼有希望在不久的将来也获得治疗*MET*基因突变的适应证。克唑替尼的常见不良反应包括视觉异常、消化道反应、肝功能异常、QT间期延长和心动过缓等。

### 3.2 卡博替尼

卡博替尼（Cabozantinib）是Ⅱ型口服MET-TKI，具有多靶点作用，包括*MET*、*VEGFR2*、*KIT*、*RET*和*AXL*。虽然卡博替尼已经上市，但其适应证是甲状腺髓样癌。初步研究结果^[27]^显示卡博替尼对NSCLC可能也有一定疗效。例如Paik等^[28]^报道1例晚期NSCLC患者同时存在*MET* 14外显子突变和*MET*扩增，应用卡博替尼治疗后肿瘤完全缓解。目前针对*MET*基因突变的Ⅱ期临床试验正在进行中。卡博替尼的不良反应主要包括腹泻、恶心呕吐等消化道反应，转氨酶升高，手足综合征，心脏毒性和高血压等。

### 3.3 沃利替尼

沃利替尼（Savolitinib, Volitinib）是Ⅰb型口服MET-TKI，由我国和记黄埔公司和阿斯利康公司合作研发。临床前研究显示沃利替尼可抑制c-MET磷酸化和下游信号传导，对于多种异种移植模型具有抗肿瘤活性，也包括*EGFR*和KRAS野生型的NSCLC。目前该药物已完成“评价沃利替尼治疗*MET*外显子14突变的局部晚期或转移性肺肉瘤样癌和其他NSCLC患者的有效性、安全性和耐受性的多中心、开放Ⅱ期的临床研究”入组。中期结果显示ORR高达51.6%（16/31），与治疗相关的不良反应大部分为1级-2级，包括：恶心、呕吐、外周组织水肿和肝功能异常^[29]^。目前沃利替尼正在进行中国食品药品监督管理局的上市申请。因此，沃利替尼可能成为在中国获批的第一款针对*MET*基因突变的靶向药物。

### 3.4 Tepotinib

Tepotinib是Ⅰb型口服MET-TKI，2020年3月在日本获批上市，用于治疗不可切除、*MET* 14外显子跳跃突变的晚期或复发性NSCLC患者。这是全球首个针对c-Met单一靶点的靶向药。其实在临床前的研究中，Tepotinib在多种肿瘤模型中都观察到抗肿瘤活性，无论MET激活是否依赖于HGF^[30]^。Tepotinib的Ⅰ期临床试验是在包括肺癌在内的实体肿瘤中进行的，着重评价具有*MET*扩增或者高表达患者的有效性和安全性^[31]^。而Tepotinib的获批是基于一项单臂、Ⅱ期VISION研究结果。该研究共纳入99例*MET*外显子14跳跃突变的NSCLC患者，接受Tepotinib治疗后的ORR达到42.4%，并且Tepotinib耐受性良好，最常见的治疗相关不良事件为周围水肿（53.8%）、恶心（23.8%）和腹泻（20.8%）^[32]^。

### 3.5 Capmatinib

Capmatinib是Ⅰb型口服MET-TKI。Capmatinib的Ⅱ期临床试验（GEOMETRY mono-1）是评估用于治疗携带*MET*外显子14跳跃突变的转移性NSCLC，包括初治患者和先前接受过治疗的患者，目前入组已结束。共97例患者入组，在初治患者中总ORR为67.9%，疾病控制率（disease control rate, DCR）为96.4%，中位无进展生存期为9.69个月。在经治患者中ORR为40.6%，DCR为78.3%，中位无进展生存期为5.42个月。受试者中约有一半的脑转移患者对Capmatinib应答（54%, 7/13）^[33, 34]^。最常见的治疗相关不良事件包括周围性水肿、恶心、肌酐升高和呕吐等。该药也已进入药品审批程序，期望在不久的将来上市。

### 3.6 Glesatinib

Glesatinib是Ⅱ型口服MET-TKI，作用靶点包括*MET*、*VEGFR*、*RON*和*TIE-2*。Ⅰ期临床试验^[35]^显示其具有较好的安全性和一定的有效性。Ⅱ期临床试验正在进行中，入组*MET*扩增和*MET* 14外显子跳跃突变的晚期NSCLC患者。

### 3.7 Merestinib

Merestinib是Ⅱ型口服MET-TKI，作用靶点包括*MET*、*TIE-1*、*AXL*、*ROS1*、*DDR1*/*2*、*FLT3*、*MERTK*、*RON*和*MKNK1*/*2*。临床前期研究^[36]^显示具有较好的抗肿瘤治疗活性。Ⅱ期临床试验正在进行中，入组*MET* 14外显子跳跃突变的晚期NSCLC患者。

## 4 作用于HGF/MET信号通路的其他药物

除了针对*MET* 14外显子突变，还有一些药物作用于HGF/MET信号通路的其他靶点。例如：抗HGF单克隆抗体（Rilotumumab, Ficlatuzumab）、抗c-Met单克隆抗体（Onartuzumab, HLX55, SHR-A1403）。这些药物都在进行临床研究中，目前尚无一种成熟药物上市。这些药物的研究多用于与MET-TKI联合，从而抑制或者逆转MET-TKI的耐药现象。另外，MET-TKI也可以与其他靶向药联合，主要作用也是抑制或者逆转耐药。例如最新发表在*The Lancet Oncology*杂志上的研究^[37]^显示，携带*EGFR*突变的晚期NSCLC患者，在接受EGFR-TKI治疗后出现耐药并伴有*MET*基因扩增时，联合应用沃利替尼和奥希替尼具有较好的疗效。因此这种联合治疗模式可能成为未来发展的方向^[38]^。

## 5 小结

*MET*基因是NSCLC的一种重要肿瘤驱动基因，与*EGFR*、*ALK*和*ROS-1*等存在互斥现象^[13]^。MET-TKI是治疗NSCLC患者中具有*MET*基因突变人群的有效药物。从已上市药物Tepotinib和即将上市的药物沃利替尼、Capmatinib的临床试验结果看，针对*MET* 14外显子的跳跃突变的单药有效率较高，ORR均达到40%以上，并且安全性较好，必将成为未来的治疗希望。但是MET-TKI的耐药不可避免，因此下一步需要加强对于MET-TKI耐药机制的研究。另外，HGF/MET信号通路的抑制剂与其他药物联合应用也可能具有治疗潜力，对于抑制和逆转耐药发挥重要作用。总之，在肺癌的精准治疗时代，分子检测和靶向药物必将为*MET*基因突变的NSCLC患者带来更好的疗效和长期生存机会。
